# Molecular Phenotyping of Patients with Sepsis and Kidney Injury and Differential Response to Fluid Resuscitation

**DOI:** 10.21203/rs.3.rs-4523416/v1

**Published:** 2024-07-02

**Authors:** Elizabeth Kiernan, Leila R. Zelnick, Ayesha Khader, Taylor D. Coston, Zoie A. Bailey, Sarah Speckmaier, Jordan Lo, Neha Sathe, Bryan R. Kestenbaum, Jonathan Himmelfarb, Nicholas Johnson, Nathan Shapiro, Ivor S. Douglas, Catherine Hough, Pavan Bhatraju

**Affiliations:** UW: University of Washington; University of Washington; University of Washington; University of Washington; University of Washington; University of Washington; University of Washington; University of Washington; University of Washington; University of Washington; University of Washington; Harvard Medical School; Denver Health Main Campus; Oregon Health & Science University; University of Washington

**Keywords:** acute kidney injury, sepsis, volume, vasopressors

## Abstract

**Purpose:**

Previous work has identified two AKI sub-phenotypes (SP1 and SP2) characterized by differences in inflammation and endothelial dysfunction. Here we identify these sub-phenotypes using biospecimens collected in the emergency department and test for differential response to restrictive versus liberal fluid strategy in sepsis-induced hypotension in the CLOVERS trial.

**Methods:**

We applied a previously validated 3-biomarker model using plasma angiopietin-1 and 2, and soluble tumor necrosis factor receptor-1 to classify sub-phenotypes in patients with kidney dysfunction (AKI or end-stage kidney disease [ESKD]). We also compared a de novo latent class analysis (LCA) to the 3-biomarker based sub-phenotypes. Kaplan-Meier estimates were used to test for differences in outcomes and sub-phenotype by treatment interaction.

**Results:**

Among 1289 patients, 846 had kidney dysfunction on enrollment and the 3-variable prediction model identified 605 as SP1 and 241 as SP2. The optimal LCA model identified two sub-phenotypes with high correlation with the 3-biomarker model (Cohen’s Kappa 0.8). The risk of 28 and 90-day mortality was greater in SP2 relative to SP1 independent of AKI stage and SOFA scores. Patients with SP2, characterized by more severe endothelial injury and inflammation, had a reduction in 28-day mortality with a restrictive fluid strategy versus a liberal fluid strategy (26% vs 41%), while patients with SP1 had no difference in 28-day mortality (10% vs 11%) (*p-value-for-interaction* = 0.03).

**Conclusion:**

Sub-phenotypes can be identified in the emergency department that respond differently to fluid strategy in sepsis. Identification of these sub-phenotypes could inform a precision-guided therapeutic approach for patients with sepsis-induced hypotension and kidney injury.

## INTRODUCTION

Acute kidney injury (AKI) is the most common form of organ failure in sepsis developing in as many as 60% in septic patients with hypotension^1,2^. Sepsis-associated AKI is associated with prolonged hospitalization, need for kidney replacement therapy, future chronic kidney disease (CKD) and death^3–8^. At present, providers base treatment decisions in AKI on parameters such as urine output and serum creatinine, yet these biomarkers fail to differentiate the highly heterogenous clinical syndrome of AKI and cannot reliably predict response to common interventions such as volume resuscitation and vasopressors in sepsis^9,10^.

We previously applied latent class analysis (LCA) to two cohorts to identify two distinct sub-phenotypes in patients with sepsis-associated AKI, and subsequently developed a parsimonious 3-variable model for clinical application^11^. This 3-variable model included plasma biomarkers of endothelial function (angiopoietin-1 (Ang-1) and angiopoietin-2 (Ang-2)) and inflammation (soluble tumor necrosis factor receptor-1 [sTNFR-1])^11^. Ang-1 and -2 are vascular endothelial growth factors that have opposing activities. Ang-1 is an agonist for the Tie-2 receptor and is protective by stabilizing the endothelium, promoting vessel maturation, and preventing microcirculatory capillary leakage. In contrast, Ang-2 typically acts as an antagonist for binding to the Tie-2 receptor and promotes endothelial dysfunction in the form of vessel destabilization, endothelial cell apoptosis, capillary leak, and deregulation of inflammation^12–14^. Independently, sTNFR-1 functions in the early innate inflammatory response in sepsis and has been associated with AKI and mortality^15^. Our model established two distinct AKI sub-phenotypes: those with a lower ratio of Ang-2/Ang-1 and low sTNFR-1, designated SP1 and characterized by endothelial stability and improved short and long-term outcomes. In contrast, those with higher ratio of Ang-2/Ang-1 and high sTNFR-1, or SP2 phenotype, were characterized by high inflammatory state and leaky endothelium and worse clinical outcomes^11,16–18^.

The Crystalloid Liberal or Vasopressors Early Resuscitation in Sepsis (CLOVERS) clinical trial randomized patients with sepsis to an initial restrictive or liberal fluid resuscitation strategy and demonstrated no differences in clinical outcomes between treatment arms or in the sub-groups with AKI or end-stage kidney disease (ESKD)^19^. Early and aggressive fluid resuscitation is a cornerstone of sepsis management which aims to improve hypoperfusion caused by increased capillary leak and endothelial permeability. In the setting of kidney injury or disease, however, the benefit of fluid resuscitation is counterbalanced by impaired volume management. We hypothesized that liberal fluid resuscitation would be preferentially harmful to septic patients with evidence of both endothelial injury and kidney disease, and that application of the SP1 or SP2 sub phenotypes may enable identification of patients most likely to benefit from early vasoconstrictive therapy. To address these questions, we first identified sub-phenotypes with known kidney injury (AKI or ESKD) using plasma samples collected in the emergency department, representing an early and critical time for treatment implementation in sepsis. Second, in a sensitivity analysis, we verified sub-phenotypes by applying LCA to 23 variables collected at study enrollment and compared sub-phenotype identification to the 3-variable model. Third, since serum creatinine can lag up to 48 hours and is insensitive for kidney injury^20^, we conducted a sensitivity analysis including patients without AKI on study enrollment to test whether the treatment effect by sub-phenotype was preserved. Fourth, to understand the overlap and differences between different sub-phenotypes in critical illness, we cross-tabulated our sub-phenotypes with a previously established hypo- and hyperinflammatory acute respiratory distress syndrome (ARDS) sub-phenotypes.

## METHODS

### Study Design and Oversight

We conducted a secondary analysis of data from CLOVERS, a multicenter, prospective, phase 3, randomized, non-blinded trial. Patients enrolled in CLOVERS were allocated to either a restrictive or liberal fluid resuscitation strategy in the setting of sepsis. All patients were randomized within 4 hours of meeting inclusion criteria, and duration of protocol for restrictive or liberal fluid resuscitation was 24 hours. Patients randomized to a restrictive fluid protocol had vasopressors prioritized as the primary treatment for sepsis-induced hypotension, with “rescue fluids” being permitted for prespecified indications that suggested severe intravascular volume depletion. The liberal fluid protocol consisted of a recommended initial 2 L IV infusion of isotonic crystalloid, followed by fluid boluses administered based on clinical triggers with “rescue vasopressors” permitted for prespecified indications. A more detailed description of methods can be found in the Supplement of the primary manuscript^19^.

### AKI Definition

AKI was defined using the KDIGO criteria as an increase in serum creatinine at randomization of ≥50% or ≥0.3 mg/dL above a baseline serum creatinine. The lowest outpatient or inpatient serum creatinine value within the last year prior to the index hospitalization was used for the baseline when available (N=909). If no pre-hospitalization serum creatinine was available and there was no known history of CKD, then we imputed a baseline serum creatinine based on an eGFR of 90 ml/min/1.73 m2 (N=517) using the 2021 creatinine-based CKD-EPI equation^11^. If no pre-hospitalization serum creatinine was available and there was a known history of CKD, then we used the lowest serum creatinine value during the index hospitalization as the baseline (N=29). Patients were subsequently staged according to the serum creatinine at randomization using the KDIGO guidelines. Seventy-five patients were on maintenance hemodialysis prior to study enrollment, (ESKD). Since ESKD patients represent severe impairment in kidney-mediated volume management and guidelines do not offer guidance on distinct therapeutic approaches across the spectrum of ESKD, we included them in the analysis.

### AKI Sub-phenotype Identification

Methods for measurement of Ang-1, Ang-2 and sTNFR-1 can be found in the online supplement. We used plasma biomarker concentrations of Ang-1, Ang-2 and sTNFR-1 to identify two sub-phenotypes (SP1 and SP2). Since the publication of our prior works, Meso Scale Discovery changed the sTNFR-1 assay from a R-Plex to a U-Plex platform. In analyses provided in the supplement we found the correlation of these two assays to be high (Pearson’s correlation*, r = 0.94*), and we created a calibration equation (**Figure S1**). We updated the 3-variable prediction model that initially used R-Plex measured sTNFR-1 to include U-Plex measured values to account for the updated sTNFR-1 biomarker platform. The model to identify AKI sub-phenotypes is TNFR_R-Plex = −101.3387 +0.3617*TNFR_U-Plex followed by the previously published prediction equation Logit(P(SP2)) = −35.44+1.99*log(Ang-2/Ang-1) + 3.41*log(TNFR_R-Plex). Participants with a probability of SP2 > 0.5 were then classified as having the SP2 sub-phenotype. Methods of LCA applied to the baseline variables can be found in the Methods in the supplement. Previously described ARDS sub-phenotypes were identified using a published four variable model that included plasma concentrations of interleukin-6, sTNFR-1, serum bicarbonate and clinical documentation of receipt of vasopressors prior to randomization^11,21^.

### Outcome Measures

The primary outcome for this analysis was 28-day and 90-day mortality. Additional outcomes included need for invasive mechanical ventilation (IMV), need for new KRT, and ICU-free days through 28 days of follow-up.

### Statistical Analysis

We compared the risk of death by sub-phenotype over 28 and 90 days of follow-up using Cox regression, adjusting in nested models for potential confounders. To test for heterogeneity of treatment effect, we completed an intention-to-treat comparison of the treatment effect of restrictive or liberal fluid resuscitation strategy on 28-day and 90-day mortality by sub-phenotype. For this analysis, we used Kaplan-Meier 28-day and 90-day mortality point estimates involving all patients who were discharged home or were still alive at day 90, with data censored at day 91. Patients who were lost to follow-up prior to day 90 were censored at the time they were last confirmed to be alive. We compared 28-day and 90-day mortality point estimates and evaluated the evidence for a treatment by sub-phenotype interaction using a non-parametric bootstrap approach with 10,000 replicates.

Secondary outcomes were the time to IMV among patients who were not receiving IMV at randomization and the time to new KRT among patients not on dialysis at the time of randomization. For these outcomes, we used a Fine-Gray sub-distribution hazard model to estimate the sub-distribution hazard ratio of each outcome accounting for the competing risk of death. Finally, we used linear regression to estimate the difference in ICU-free days through day 28 between the two treatment groups. All analyses were conducted using the R 4.2.3 software environment (R Foundation for Statistical Computing, Vienna, Austria). Two-sided p-values < 0.05 were taken as evidence of statistical significance.

## RESULTS

### Baseline Characteristics of Participants by Sepsis-associated AKI Sub-phenotype

**Figure 1:**
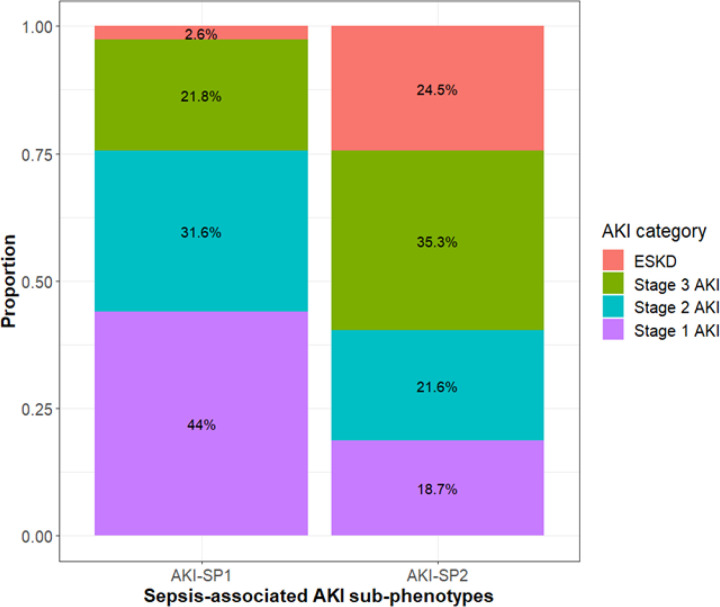
Makeup of sub-phenotypes by proportion of KDIGO Stage of AKI and ESKD Provided is the proportion of participants with Kidney Disease Improving Global Outcomes (KDIGO) Stage 1, 2 or 3 AKI and end-stage kidney disease (ESKD) within each sub-phenotype. Participants with SP2 had a higher proportion of participants with more severe AKI compared to participants with SP1.

Among 1563 patients enrolled in CLOVERS, 1289 had biospecimens collected prior to randomization and available for analysis (Figure 1). Among the 1289 patients, 846 (66%) had kidney dysfunction (771 had AKI and 75 had ESKD) at study enrollment and 443 (34%) did not have AKI or ESKD. Among the 846 patients with kidney dysfunction the previously published sub-phenotype classification model, incorporating plasma Ang-1, Ang-2, and sTNFR-1 measurements at randomization, classified 605 (72%) patients as SP1 and 241 (28%) as SP2 (Table 1). As expected by randomization, the distribution of SP1 and SP2 were similar between treatment arms. Patients with SP2 had more severe KDIGO stage of AKI on study enrollment compared to SP1 (**Figure S2**).

To determine whether SP2 is enriched in the AKI population, we applied the 3-biomarker prediction model to patients without AKI or ESKD on study enrollment. Among 443 patients, we found that 411 (93%) were classified as SP1 and 32 (7%) as SP2. The 32 patients with SP2 were characterized by high rates of CKD and ten patients subsequently developed AKI within 48 hours of study enrollment based on changes in serum creatinine (**Table S1**). In a sensitivity analysis, we applied LCA to 23-variables collected at study enrollment and found that a 2-class model best separated the data (**Table S3**). We also found high correlation between the LCA derived sub-phenotypes and the 3-variable biomarker sub-phenotypes, Cohen’s Kappa of 0.80. With the high correlation and to support prospective identification of these sub-phenotypes, we used the 3-variable derived sub-phenotypes for the subsequent analyses.

### Clinical Outcomes by Sub-Phenotype

To evaluate the association of sub-phenotypes with clinical outcomes, patients without AKI were compared against those with SP1 and SP2 and then patients with SP1 and SP2 were directly compared (**Table S4**). In models adjusting for demographics, comorbidities, KDIGO stage of AKI or ESKD and baseline sequential organ failure assessment (SOFA) scores, patients with SP1 had a similar risk of new KRT, ICU length of stay, receipt of IMV, 28-day and 90-day mortality as patients with no AKI. Patients with SP2 had greater risk of 28-day (HR = 2.33 (95% CI: 1.60, 3.38) and 90-day mortality (HR = 1.98 (95% CI: 1.45, 2.71) than SP1. Patients with SP2 were more likely than those with SP1 to require new KRT (sHR=3.03 (95% CI: 1.42, 6.47) or IMV after study enrollment (sHR=1.54 (95% CI: 1.00, 2.37) (**Table S5**). Those with SP2 had ICU stays that were 3 days longer than those with SP1 (adjusted difference 3.1 days (95% CI: 1.8 to 4.5 days) (**Table S7**).

### Difference in Volume of Fluid and Timing of Vasopressors by CLOVERS Randomization

In the 24-hour protocol following randomization, patients with SP1 randomized to the liberal resuscitation strategy received on average 2 L more of IV fluids than patients with SP1 randomized to the restrictive resuscitation strategy (3,684 ± 1,644 mL vs 1,684 ± 1657 mL) (**Table S6**). Similarly, patients with SP2 randomized to the liberal resuscitation strategy received on average 4,091 (± 1793) mL compared to 2,244 (± 2,305) mL in the restrictive strategy. Consistent with the trial protocol, patients randomized to the restrictive strategy received vasopressors more often than patients in the liberal strategy across both sub phenotypes.

### Interaction Between Sub-phenotype and Resuscitation Strategy

**Figure 2:**
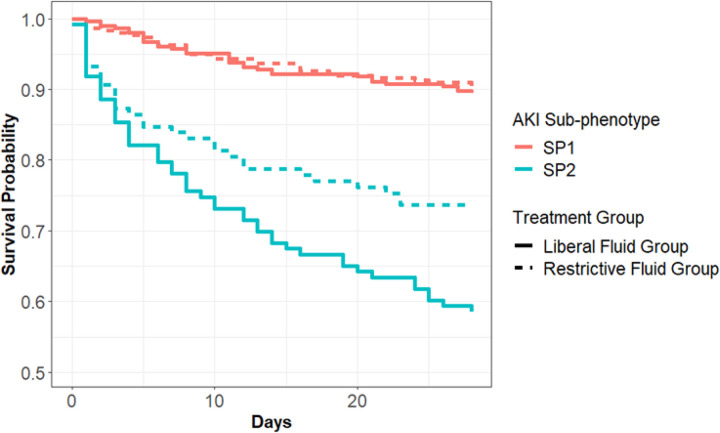
Kaplan-Meier survival curve in sepsis-associated AKI sub-phenotypes stratified by resuscitation treatment group Curves for each sub-phenotype are stratified by liberal or restrictive resuscitation strategy.

**Figure 3:**
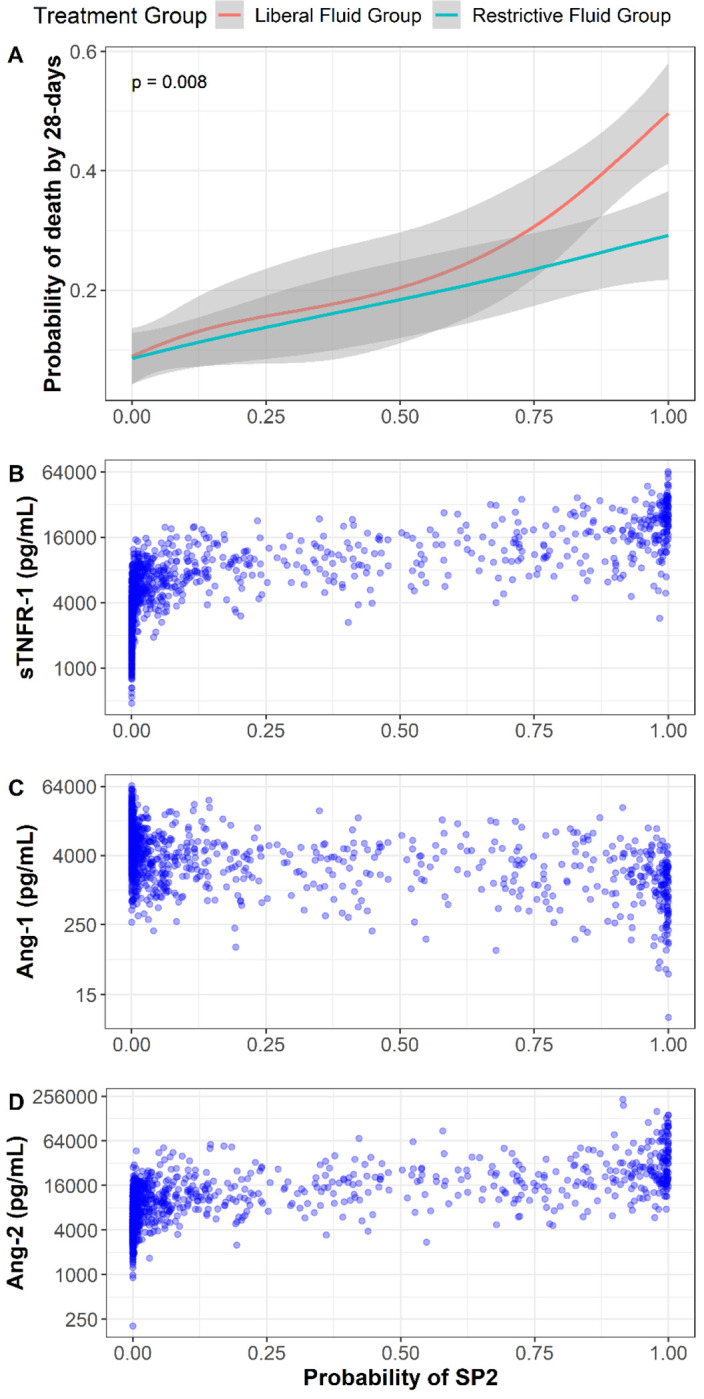
Probability belonging to SP-2 and differential treatment response to a restrictive and liberal fluid resuscitation strategy Panel A is the probability of belonging to SP2 (x-axis) and the probability of 28-day mortality (y-axis). The lines plot the estimated mortality in either a liberal (red) or restrictive (blue) resuscitation strategy with 95% CIs over a range of probabilities of assignment to SP2. The p-value tests the null hypothesis of no interaction between treatment group and the continuous probability of SP2 membership, and excludes participants who were censored prior to day 28. Plots B, C and D below demonstrate the concentrations of sTNFR-1, Ang-1 and Ang-2 concentrations at different probabilities of belonging to SP2.

Next, we determined whether sub-phenotypes identified using biospecimens collected prior to randomization respond differently to the fluid resuscitation strategy. In SP1, a similar proportion of patients died at 28 days with liberal and restrictive fluid strategies (11% vs 10%, respectively) (Figure 2). In SP2 a liberal strategy compared with a restrictive strategy led to greater 28-day mortality (41% vs 26% respectively) and the difference in treatment effects between SP1 and SP2 was 14% (95% CI: 2%, 27%); *p-value for interaction = 0.03*. This effect was not observed in the patients without AKI (Table 2). Among patients with SP1, 90-day mortality with a liberal and restrictive fluid strategies was 18% versus 17%, while SP2 was 48% versus 26% (p-value for interaction = *0.06*). We did not observe a difference for alternative outcomes among sub-phenotypes by treatment (**Table S8**). Next, we evaluated the continuous probability of belonging to SP2 and the interaction of a liberal versus a restrictive fluid resuscitation. We observed a significant interaction between the probability of sub-phenotype and fluid resuscitation strategy and 28-day mortality (*p=0.008*) (Figure 3). As patients had a greater certainty of the SP2 phenotype (i.e. higher probability of SP2 based on the 3-biomarker model), we observed a larger difference in 28-day mortality between a liberal versus restrictive fluid resuscitation strategy. In the primary CLOVERS publication, there was no observed heterogeneity of treatment effect based on SOFA scores or ESKD status^19^.

In sensitivity analyses, we evaluated sub-phenotypes restricted to the AKI population and excluded ESKD. Among patients with SP1, 28-day mortality with liberal and restrictive fluid strategies was 10% versus 10% and in patients with SP2 was 39% versus 29% (p-value for interaction = 0.16) (**Table S9**). In another sensitivity analysis, we used the 3-biomarker model to classify all patients with plasma available in CLOVERS as SP1 and SP2. Among patients with SP1, 28-day mortality with a liberal and restrictive fluid strategies was 9% versus 9% and in patients with SP2 was 41% versus 27% (p-value for interaction = *0.02*). We also observed a similar decrease in 90-day mortality in SP2 (Table 2).

### Cross-Tabulation of AKI and ARDS Sub-phenotypes

We directly compared the cross-tabulation of SP1 and SP2 and ARDS sub-phenotypes (hypoinflammatory and hyperinflammatory). Cross-tabulation demonstrated mild to moderate overlap between hypoinflammatory and SP1 and hyperinflammatory and SP2 but also demonstrated distinct differences (Cohen’s Kappa for Agreement of 0.42) (**Table S10**). Out of 846 patients, 77.0% were concordant (i.e. hypoinflammatory and SP1 or hyperinflammatory and SP2), and 23.0% were classified discordantly. We observed no differential response to a restrictive or liberal fluid resuscitation strategy between ARDS sub-phenotypes for 28-day (p-value for interaction = 0.25) or 90-day mortality (p-value for interaction = 0.60) (**Table S11**).

## DISCUSSION

The heterogeneity of AKI has limited insight into clinical trajectory and hindered development of interventions that target an individual patient’s pathophysiology^22–25^. We completed a secondary analysis of the CLOVERS trial and demonstrated that sub-phenotypes previously defined in ICU patients were also present in the emergency department, an earlier and critical window for therapeutic decisions in sepsis treatment. These sub-phenotypes predicted differential treatment response to liberal versus restrictive resuscitation strategies among patients with kidney dysfunction; revealing a treatment effect which was not seen in the original CLOVERS study. Taken together, these data offer a precision medicine treatment strategy in sepsis-induced hypotension and kidney dysfunction based on a 3-biomarker prediction model which may identify populations with differential response to fluid resuscitation strategy.

Despite a long-held belief by clinicians that a rise in serum creatinine early during sepsis should result in additional fluid therapy^9^, patients with SP2 and kidney dysfunction had a greater mortality with a liberal fluid strategy compared to a restrictive fluid strategy. A difference in clinical outcomes between fluid strategy was not observed in patients without AKI/ESKD or patients with SP1. While these findings are hypothesis-generating, it suggests that early in sepsis the combination of kidney dysfunction and endothelial injury may identify a sub-phenotype that is harmed by a liberal fluid therapy. In addition, the observed heterogeneity of treatment effect was not present when identifying sub-groups based on SOFA scores, ESKD or AKI status^26,27^, suggesting that the sub-phenotypes capture information that is not readily available by clinical definitions. In this manner, application of the 3-biomarker prediction model to all patients with sepsis-induced hypotension may improve generalizability and time to sub-phenotype identification to facilitate future clinical trials without misclassification of patients with SP2.

Our prior work leveraged these AKI sub-phenotypes in the Vasopressin and Septic Shock Trial (VASST), a randomized control trial investigating early addition of vasopressin to norepinephrine in sepsis. In that analysis, we showed that in SP1, early addition of vasopressin compared to norepinephrine alone was associated with improved 90-day mortality, but in SP2, vasopressin showed no significant treatment difference. Extrapolating these findings with the current CLOVERS analysis, we hypothesize that patients with the SP1 would derive benefit from either a restrictive or liberal fluid resuscitation and early addition of vasopressin with norepinephrine for persistent hypotension, while those with the SP2 may benefit from an initial restrictive fluid strategy and monotherapy with norepinephrine.

Our study has several strengths. First, a majority of patients had a pre-hospitalization serum creatinine value available to approximate baseline kidney function and to accurately determine AKI status early after hospital presentation. Leveraging this data highlighted that approximately two-thirds of patients in the trial had AKI on hospital presentation for sepsis-induced hypotension. These findings are consistent with prior studies of AKI in septic shock and highlight that prevention of AKI in sepsis may be infeasible and therapies that target recovery should be prioritized^28,29^. Second, we were able to leverage the randomization in the CLOVERS trial to address limitations of indication bias of fluid administration in observational studies to demonstrate that patients with SP2, characterized by greater endothelial dysfunction and inflammation, have an improved mortality with a restrictive resuscitation strategy compared to a liberal strategy. Third, the CLOVERS trial included patients with ESKD, a group that is often excluded from large randomized clinical trials^30^. While limited by small group size, our data suggest that most patients with ESKD have a sub-phenotype consistent with SP2 (79%) and may benefit from a restrictive fluid resuscitation strategy.

This analysis should be interpreted in the context of its limitations. First, there was variation in the amount of fluid received in the CLOVERS trial in the restrictive and liberal fluid groups and overlap of total volumes between groups may dampen a potential therapeutic signal. Second, this was a retrospective study of a completed clinical trial. However, the study design and prediction model for sub-phenotype identification were defined a *priori*. Third, we underscore that our work does not preclude that alternative AKI sub-phenotypes are present. Fourth, elevations of angiopoietins and sTNFR-1 are not specific to AKI. However, a large body of preclinical and clinical studies have demonstrated the importance of these pathways and biomarkers in kidney diseases, such as AKI and CKD^13,31,32^. Fifth, serum creatinine concentrations were not collected after day 3 of participation in CLOVERS and so we are unable to draw conclusions about differential rate of kidney recovery between sub-phenotypes.

In summary, this work posits that early identification of AKI sub-phenotypes can inform clinical decision making in patients with sepsis-induced hypotension. We observed that SP2 had improved 28-day mortality with a restrictive fluid resuscitation strategy. Identification of AKI sub-phenotypes may facilitate development of targeted treatment interventions in critically ill patients.

## Figures and Tables

**Table 1.** Baseline characteristics stratified by sub-phenotypes.

	No AKI (N = 443)	SP1 (N = 605)	SP2 (N = 241)
**Age (years), average ± SD**	56.1 ± 17.0	61.7 ± 14.6	62.7 ± 15.1
**Male, n (%)**	217 (49%)	332 (55%)	133 (55%)
**Race, n (%)**			
White	327 (74%)	435 (72%)	158 (66%)
Black	51 (12%)	100 (17%)	55 (23%)
Asian	18 (4%)	15 (2%)	8 (3%)
Other	6 (1%)	5 (1%)	2 (1%)
Not reported	41 (9%)	50 (8%)	18 (7%)
**Ethnicity, n (%)**			
Hispanic/Latino	74 (17%)	88 (15%)	28 (12%)
Not Hispanic/Latino	353 (80%)	493 (81%)	207 (86%)
Not reported	16 (4%)	24 (4%)	6 (2%)
**Randomization location, n (%)**			
ED	412 (93%)	566 (94%)	209 (87%)
ICU	21 (5%)	38 (6%)	30 (12%)
Other	10 (2%)	1 (0%)	2 (1%)
**Invasive mechanical ventilation, n (%)**	26 (6%)	35 (6%)	21 (9%)
**Source of infection, n (%)**			
Pneumonia	152 (34%)	163 (27%)	55 (23%)
Urinary tract infection	83 (19%)	168 (28%)	57 (24%)
Intra-abdominal infection	43 (10%)	55 (9%)	31 (13%)
Skin or soft tissue infection	57 (13%)	54 (9%)	21 (9%)
Other or unknown	108 (24%)	165 (27%)	77 (32%)
**Weight (kg), average ± SD**	75.1 ± 26.9	80.7 ± 24.9	80.9 ± 24.4
**BMI (kg/m2), average ± SD**	26.3 ± 8.2	28.1 ± 7.8	28.2 ± 8.6
**SOFA score, average ± SD**	2.2 ± 2.2	3.4 ± 2.3	5.7 ± 3.0
**History of CKD, n (%)**	19 (4%)	46 (8%)	74 (31%)
**Baseline serum creatinine (mg/dL), average ± SD**	0.9 ± 0.8	0.8 ± 0.4	0.9 ± 0.4
**Randomization serum creatinine (mg/dL), average ± SD**	0.9 ± 0.8	2.0 ± 1.5	3.6 ± 2.5
**Volume of fluid administered before randomization (mL), median (IQR)**	2000 (1400–2400)	2100 (1525–2500)	2100 (1450–2498)
**Biomarker Concentrations, median (IQR)**			
sTNFR-1	3207 (2062–5464)	5391 (3713–8333)	18553 (11899–27238)
Ang-1	5442 (2450–9743)	5477 (2997–9360)	1768 (801–3953)
Ang-2	6053 (4093–10531)	8518 (5315–13138)	23311 (15949–38385)
Ang-2/Ang-1 ratio	1.2 (0.6–3.1)	1.6 (0.8–3.4)	13.6 (7.4–27.8)

Entries are mean (SD) for continuous variables and N (%) for categorical variables, except as noted.

Abbreviations: BMI, body mass index; SOFA, sequential organ failure assessment scores; CKD, chronic kidney disease; sTNFR-1, soluble tumor necrosis factor receptor-1; Ang-1, angiopoietin-1; Ang-2, angiopoietin-2

**Table 2.** Treatment effects by sub-phenotype status among patients with kidney dysfunction and allpatients in CLOVERS

CLOVERS Populations		% who died, liberal	% who died, restrictive	Difference, restrictive – liberal (95% CI)	Difference of Difference (95% CI)	p-value for interaction

**Patients with AKI and ESKD (n=846)**						

28-day mortality	SP-1	11%	10%	−1% (−6%, 4%)	14% (2%, 27%)	0.03

	SP-2	41%	26%	−15% (−27%, −3%)		

90-day mortality	SP-1	18%	17%	−1% (−7%, 5%)	13% (−1%, 27%)	0.06

	SP-2	48%	36%	−13% (−24%, −1%)		

**Patients without AKI or ESRD (n=443)**						

28-day mortality	SP-1	6%	8%	2% (−3%, 6%)	2% (−33%, 38%)	0.89

	SP-2	36%	35%	−1% (−36%, 34%)		

90-day mortality	SP-1	11%	16%	5% (−2%, 11%)	7% (−30%, 43%)	0.72

	SP-2	43%	41%	−2% (−38%, 34%)		

**All Patients (n=1289)**						

28-day mortality	SP-1	9%	9%	0% (−3%, 4%)	14% (2%, 25%)	0.02

	SP-2	41%	27%	−13% (−25%, −2%)		

90-day mortality	SP-1	15%	17%	1% (−3%, 6%)	14% (1%, 27%)	0.03

	SP-2	48%	36%	−13% (−24%, −1%)		
		

## Data Availability

Data available within the article or its supplementary materials

## Take Home Message

We applied a 3 biomarker prediction model to the CLOVERS analysis and showed feasbility of identifying sub-phenotypes of patients with sepsis. These sub-phenotypes are clinically distinct and respond differently to fluid resuscitation in sepsis. Identification of sub-phenotypes could inform precision-guided therapy for patients with sepsis-associated hypotension and kidney injury.

## References

[1] Bhatraju PK, Robinson-Cohen C, Mikacenic C, Circulating levels of soluble Fas (sCD95) are associated with risk for development of a nonresolving acute kidney injury subphenotype. Crit Care. 2017;21(1). doi:10.1186/S13054-017-1807-X PMC5559814 28814331

[2] Kellum JA, Chawla LS, Keener C, The Effects of Alternative Resuscitation Strategies on Acute Kidney Injury in Patients with Septic Shock. Am J Respir Crit Care Med. 2016;193(3):281–287. doi:10.1164/RCCM.201505-0995OC 26398704 PMC4803059

[3] Joannidis M, Metnitz PGH. Epidemiology and natural history of acute renal failure in the ICU. Crit Care Clin. 2005;21(2):239–249. doi:10.1016/J.CCC.2004.12.005 15781160

[4] Hoste EAJ, Bagshaw SM, Bellomo R, Epidemiology of acute kidney injury in critically ill patients: the multinational AKI-EPI study. Intensive Care Med. 2015;41(8):1411–1423. doi:10.1007/S00134-015-3934-7 26162677

[5] Clermont G, Acker CG, Angus DC, Sirio CA, Pinsky MR, Johnson JP. Renal failure in the ICU: comparison of the impact of acute renal failure and end-stage renal disease on ICU outcomes. Kidney Int. 2002;62(3):986–996. doi:10.1046/J.1523-1755.2002.00509.X 12164882

[6] Bellomo R, Kellum JA, Ronco C. Acute kidney injury. Lancet (London, England). 2012;380(9843):756–766. doi:10.1016/S0140-6736(11)61454-2 22617274

[7] Bellomo R, Ronco C, Kellum JA, Mehta RL, Palevsky P. Acute renal failure - definition, outcome measures, animal models, fluid therapy and information technology needs: the Second International Consensus Conference of the Acute Dialysis Quality Initiative (ADQI) Group. Crit Care. 2004;8(4). doi:10.1186/CC2872 PMC522841 15312219

[8] Uchino S, Kellum JA, Bellomo R, Acute renal failure in critically ill patients: a multinational, multicenter study. JAMA. 2005;294(7):813–818. doi:10.1001/JAMA.294.7.813 16106006

[9] Moledina DG, Parikh CR. Phenotyping of Acute Kidney Injury: Beyond Serum Creatinine. Semin Nephrol. 2018;38(1):3–11. doi:10.1016/J.SEMNEPHROL.2017.09.002 29291759 PMC5753429

[10] Bellomo R, Kellum JA, Ronco C, Acute kidney injury in sepsis. Intensive Care Med. 2017;43(6):816–828. doi:10.1007/s00134-017-4755-7 28364303

[11] Bhatraju PK, Zelnick LR, Herting J, Identification of Acute Kidney Injury Subphenotypes with Differing Molecular Signatures and Responses to Vasopressin Therapy. Am J Respir Crit Care Med. 2019;199(7):863–872. doi:10.1164/RCCM.201807-1346OC 30334632 PMC6444649

[12] Bhatraju PK, Cohen M, Nagao RJ, Genetic variation implicates plasma angiopoietin-2 in the development of acute kidney injury sub-phenotypes. BMC Nephrol. 2020;21(1). doi:10.1186/S12882-020-01935-1 PMC7368773 32680471

[13] Mansour SG, Bhatraju PK, Coca SG, Angiopoietins as Prognostic Markers for Future Kidney Disease and Heart Failure Events after Acute Kidney Injury. J Am Soc Nephrol. 2022;33(3):613–627. doi:10.1681/ASN.2021060757 35017169 PMC8975075

[14] Samuel CS. Targeting angiopoietin-2 as a novel treatment option for kidney fibrosis. Kidney Int. 2022;102(4):691–694. doi:10.1016/J.KINT.2022.07.023 36150760

[15] Mikacenic C, Hahn WO, Price BL, Biomarkers of Endothelial Activation Are Associated with Poor Outcome in Critical Illness. PLoS One. 2015;10(10). doi:10.1371/JOURNAL.PONE.0141251 PMC4619633 26492036

[16] Li Y, Liu P, Zhou Y, Activation of Angiopoietin-Tie2 Signaling Protects the Kidney from Ischemic Injury by Modulation of Endothelial-Specific Pathways. J Am Soc Nephrol. 2023;34(6):969–987. doi:10.1681/ASN.0000000000000098 36787763 PMC10278803

[17] Star BS, Boahen CK, van der Slikke EC, Plasma proteomic characterization of the development of acute kidney injury in early sepsis patients. Sci Rep. 2022;12(1). doi:10.1038/S41598-022-22457-W PMC9668831 36385130

[18] Wiersema R, Jukarainen S, Vaara ST, Two subphenotypes of septic acute kidney injury are associated with different 90-day mortality and renal recovery. Crit Care. 2020;24(1). doi:10.1186/S13054-020-02866-X PMC7161019 32295614

[19] NI S IS D, RG B, Early Restrictive or Liberal Fluid Management for Sepsis-Induced Hypotension. N Engl J Med. 2023;388(6):499–510. doi:10.1056/NEJMOA2212663 36688507 PMC10685906

[20] Zhou H, Hewitt SM, Yuen PST, Star RA. Acute Kidney Injury Biomarkers-Needs, Present Status, and Future Promise NIH Public Access. Nephrol Self Assess Progr. 2006;5(2):63–71. PMC2603572 19096722

[21] Sinha P, Delucchi KL, Chen Y, Latent class analysis-derived subphenotypes are generalisable to observational cohorts of acute respiratory distress syndrome: a prospective study. Thorax. 2022;77(1):13–21. doi:10.1136/THORAXJNL-2021-217158 34253679 PMC8688287

[22] Joannidis M, Metnitz PGH. Epidemiology and natural history of acute renal failure in the ICU. Crit Care Clin. 2005;21(2):239–249. doi:10.1016/J.CCC.2004.12.005 15781160

[23] Hoste EAJ, Bagshaw SM, Bellomo R, Epidemiology of acute kidney injury in critically ill patients: the multinational AKI-EPI study. Intensive Care Med. 2015;41(8):1411–1423. doi:10.1007/S00134-015-3934-7 26162677

[24] Ronco C, Bellomo R, Kellum JA. Acute kidney injury. Lancet (London, England). 2019;394(10212):1949–1964. doi:10.1016/S0140-6736(19)32563-2 31777389

[25] Clermont G, Acker CG, Angus DC, Sirio CA, Pinsky MR, Johnson JP. Renal failure in the ICU: Comparison of the impact of acute renal failure and end-stage renal disease on ICU outcomes. Kidney Int. 2002;62(3):986–996. doi:10.1046/j.1523-1755.2002.00509.x 12164882

[26] Khader A, Zelnick LR, Sathe NA, The Interaction of Acute Kidney Injury with Resuscitation Strategy in Sepsis: A Secondary Analysis of a Multicenter, Phase 3, Randomized Trial (CLOVERS). Am J Respir Crit Care Med. October 2023. doi:10.1164/RCCM.202308-1448LE PMC10765399 37870416

[27] NI S IS D, RG B, Early Restrictive or Liberal Fluid Management for Sepsis-Induced Hypotension. N Engl J Med. 2023;388(6):499–510. doi:10.1056/NEJMOA2212663 36688507 PMC10685906

[28] Reynolds PM, Stefanos S, MacLaren R. Restrictive resuscitation in patients with sepsis and mortality: A systematic review and meta-analysis with trial sequential analysis. Pharmacotherapy. 2023;43(2):104–114. doi:10.1002/phar.2764 36625778 PMC10634281

[29] Manrique-Caballero CL, Del Rio-Pertuz G, Gomez H. Sepsis-Associated Acute Kidney Injury. Crit Care Clin. 2021;37(2):279–301. doi:10.1016/J.CCC.2020.11.010 33752856 PMC7995616

[30] Chewcharat A, Chang YT, Sise ME, Bhattacharyya RP, Murray MB, Nigwekar SU. Phase-3 Randomized Controlled Trials on Exclusion of Participants With Kidney Disease in COVID-19. Kidney Int reports. 2021;6(1):196–199. doi:10.1016/J.EKIR.2020.10.010 PMC7577277 33106779

[31] Coca SG, Vasquez-Rios G, Mansour SG, Plasma Soluble Tumor Necrosis Factor Receptor Concentrations and Clinical Events After Hospitalization: Findings From the ASSESS-AKI and ARID Studies. Am J Kidney Dis. 2023;81(2):190–200. doi:10.1053/J.AJKD.2022.08.007 36108888 PMC9868060

[32] Niewczas MA, Pavkov ME, Skupien J, A signature of circulating inflammatory proteins and development of end-stage renal disease in diabetes. Nat Med. 2019;25(5):805–813. doi:10.1038/S41591-019-0415-5 31011203 PMC6508971

